# Silencing of Unintegrated Retroviral DNAs

**DOI:** 10.3390/v13112248

**Published:** 2021-11-09

**Authors:** Stephen P. Goff

**Affiliations:** Department of Biochemistry and Molecular Biophysics, Columbia University Medical Center, New York, NY 10032, USA; spg1@cumc.columbia.edu

**Keywords:** unintegrated retroviral DNA, transcriptional silencing, histone modification

## Abstract

Retroviral infection delivers an RNA genome into the cytoplasm that serves as the template for the synthesis of a linear double-stranded DNA copy by the viral reverse transcriptase. Within the nucleus this linear DNA gives rise to extrachromosomal circular forms, and in a key step of the life cycle is inserted into the host genome to form the integrated provirus. The unintegrated DNA forms, like those of DNAs entering cells by other means, are rapidly loaded with nucleosomes and heavily silenced by epigenetic histone modifications. This review summarizes our present understanding of the silencing machinery for the DNAs of the mouse leukemia viruses and human immunodeficiency virus type 1. We consider the potential impact of the silencing on virus replication, on the sensing of the virus by the innate immune system, and on the formation of latent proviruses. We also speculate on the changeover to high expression from the integrated proviruses in permissive cell types, and briefly consider the silencing of proviruses even after integration in embryonic stem cells and other developmentally primitive cell types.

## 1. Introduction

The introduction of foreign DNA into cells typically triggers an array of responses analogous to the sounding of alarms—the DNA is treated as a warning sign that something unusual and potentially dangerous to the cell has occurred. Exogenous DNA in the cytoplasm is detected by a number of sensors, including cGAS (cGAS), Ku70, IFI16, and others, which activate pathways leading to the production of interferons and the induction of an antiviral state in the target cell, as well as in its neighboring cells [1,2,3]. In a second layer of defense, any DNA that finds its way into the nucleus is subject to tight transcriptional silencing. This function seems likely to have evolved to inhibit or restrict infection by viruses that would otherwise initiate a course of viral gene expression, viral replication, and potentially cell death [4]. These events have been most heavily studied in the context of infection by DNA viruses, such as the herpes viruses, but they clearly act in many diverse settings. In particular, the process has been found to act to potently block transcription of the viral DNAs formed in the early hours after infection of most cells by a number of retroviruses. These DNAs, generated by reverse transcription of the incoming viral RNA genome, consist of unintegrated double-stranded DNAs in a mixture of conformations, resident in a large, poorly characterized complex dubbed the “preintegration complex” (or PIC). These unintegrated DNAs are only very weakly transcribed in most cell types—they are heavily silenced [5,6]. The integration of the viral DNA, establishing the integrated provirus, dramatically relieves this silencing and most often triggers the high-level transcription of the DNA, producing both viral genomic RNA and a collection of viral mRNAs that are translated to give rise to viral proteins and ultimately progeny virions. This chapter will review what we know now about the events of repressing unintegrated retroviral DNA expression and the cellular machinery involved in mediating the silencing of these DNAs. We concentrate on work from our own laboratory but include recent findings from others in the field. We suggest that all retroviral DNAs are not repressed by the identical host factors, but rather that partially overlapping sets of factors act on various members of the retrovirus family, which are known to enter into the nucleus via very distinctive routes. We will speculate on the mechanisms of action of the changes that occur upon integration of the DNA into the host genome.

## 2. Repression of Incoming DNAs: Early Evidence for the Involvement of Chromatin and Histone Modifications

In the earliest days of transformation of mammalian cells in culture by the introduction of DNAs, the efficiency of this process was low, and this was accepted as unsurprising [7,8]. There were many reasons to expect this to be the case—DNA was not efficiently taken up by cells, not efficiently transferred into the nucleus, and not efficiently incorporated into chromosomal DNA. Typically, very large amounts of DNA were applied to achieve either the transient expression of DNA constructs or stable expression of the DNA associated with its incorporation into the host genome [9]. Methods such as calcium phosphate coprecipitation [10], complexing DNA with DEAE dextran [11], encasing DNA in lipid vesicles, microinjection, and electroporation all improved the efficiency, but high levels of DNA were still typically required for successful DNA delivery. Introduction of DNA genomes of DNA viruses, such as SV40, a process dubbed transfection, suffered from similar inefficiencies.

Studies of the state of transfected viral DNAs revealed that the DNAs introduced into mammalian cells with calcium phosphate or DEAE Dextran were rapidly assembled into “minichromosomes” containing nucleosomes [12], arranged very much like the nucleosomes in chromosomal DNA and in virion particles [13]. These nucleosomes showed the typical spacing of roughly 190 bp along the viral DNA [14], though perhaps not as well-ordered as in chromosomes. The loading of histones onto the transfected DNAs was observed in both replicating and non-replicating DNAs, indicating that the process was not necessarily coupled to DNA replication. There were indications that these histones had high relevance to the expression of the incoming DNA. In the search for methods to enhance the efficiency of transformation and transfection, it was noted that any of a number of histone deacetylase inhibitors could profoundly stimulate the expression of transfected DNAs [15,16]—including sodium butyrate, and later the more potent and broad-spectrum HDAC inhibitor Trichostatin A (TSA) [17]. TSA stimulates gene expression in many settings [16,18,19], and though the effects on transfected DNA expression could have been indirect—acting by altering cellular expression profiles—it was supposed that the histones loaded on the transfected DNA might be the direct targets of these compounds. The results suggested that expression of transfected DNA was limited by the deacetylation of histones loaded onto the incoming DNAs, as known in other settings [20,21]. Examination of the state of the incoming DNA supported this idea. Treatment of transfected cells with sodium butyrate for a short period of time immediately following transfection markedly increased the DNase I digestion sensitivity of the newly assembled plasmid chromatin [12], reflecting increased opening or accessibility of the DNA. Furthermore, minichromosomes isolated from such butyrate-treated cells were depleted of histone H1 and contained highly acetylated forms of histone H4, characteristics of transcriptionally active chromatin structures.

The silencing of incoming DNA is a feature of infection by many viruses. A large body of work on the herpes viruses has shown that the incoming DNA is rapidly formed into chromatin by the addition of nucleosomes [22,23]. HSV DNA in the virion particle is not associated with histones but is condensed into heterochromatin upon entry into the cell [24]. The viruses have been able to counteract this using the activities of various viral proteins. The HSV-1 gene products VP16 and ICP0 can remove repressive histones and enhance their acetylation to promote viral DNA expression and replication [25,26], and the importance of this function is indicated by the fact that ICP0-null viruses are subject to profound epigenetic silencing. There is evidence that the histone chaperones ATRX/DAXX play a major role in mediating the silencing [27]. Though the replication cycle of retroviruses is profoundly different from that of the DNA viruses, these viruses too are subject to similar host silencing.

Retroviral DNAs are distinctive from other viral DNA genomes in that they are not actually delivered into cells by incoming virions but rather are synthesized de novo from the incoming viral RNA, and are therefore completely “virgin”—never having been previously exposed to any host factors. Retroviral infection of permissive cells results in reverse transcription of the viral RNA genome to form a linear double-stranded DNA that is subsequently trafficked into the nucleus [28,29]. The process of reverse transcription takes place in the context of a large protein complex closely resembling the intact virion core, termed the “reverse transcription complex” or RTC, and when completed, gives rise to the “pre-integration complex” or PIC. After entry into the nucleus, the linear DNA gives rise to several additional DNA forms: two circular forms, containing either one or two LTRs, and the integrated proviral DNA. There has been tremendous uncertainty, and much debate, as to where in the cells and when these events take place. Our view is that the answers to these contentious issues will not be identical for all retroviruses, but rather that there will be profound differences among the various retroviruses. The timing and location of viral DNA synthesis, and the timing relative to nuclear entry, may well also depend on the cell types being infected.

The two most studied retroviruses with respect to nuclear entry are the Moloney murine leukemia virus (MLV), a so-called “simple retrovirus” and member of the gammaretrovirus family, and the human immunodeficiency virus type 1 (HIV-1), a member of the lentivirus family. It is clear that the requirements in the physiological state of the cell for successful infection by MLV and HIV-1 are dramatically different, and this may affect the location and timing of reverse transcription. MLV exhibits a strict requirement for mitosis for replication [30], and infection of postmitotic, nondividing cells, or cells arrested in the cell cycle by addition of aphidicolin, results in the reverse transcription of the genome in the cytoplasm, but no formation of nuclear forms. The DNA formed in the arrested cells can be full-length and the resulting PICs are competent for integration in vitro, indicating that reverse transcription for MLV, at least in this setting, is completed in the cytoplasm. The MLV PIC apparently cannot enter the intact nucleus, but rather must wait for nuclear breakdown during mitosis to gain access to the host chromosomes and establish the integrated provirus. HIV-1, in contrast, can infect nondividing cells as efficiently as rapidly dividing cells [31,32,33], and HIV-1 cores have recently been shown capable of entry into intact nuclei through nuclear pores [34,35,36]. Indeed, it is even possible that it only poorly infects cells without passage through pores of intact nuclei [37]. There are likely more than one route of entry [38], largely determined by the viral capsid (CA) protein. The existence of these alternative pathways has been most clearly revealed by the distinctive requirements for host factors exhibited by different mutant CA proteins [39]. Finally, we note that HIV-1 reverse transcription may begin in the cytoplasm, but it has been recently revealed that it is completed only within the nucleus, after import of the RTC into the nucleus, and perhaps only very briefly before the time of DNA integration [40]. Full uncoating and release of the viral DNA occurs in the nucleus [41].

In spite of the very different times and locations of reverse transcription, and the very different routes of entry into the nucleus, MLV and HIV-1 are both subject to profound silencing of their unintegrated DNA forms once they have entered the nucleus. The silencing of the viruses is similar in many regards, as we will see, but does not require identical host factors. We will here review separately the silencing of MLV and HIV-1 unintegrated DNAs.

## 3. Silencing of Unintegrated MLV DNA

It has long been known that unintegrated retroviral DNAs are very poorly expressed, and that high-level expression occurs only after integration of the DNA into the host genome [5,6,42,43,44]. One early proposal was that the loading of nucleosomal histones onto the DNA might be required for efficient transcription and that the unintegrated viral DNA had not yet acquired nucleosomes. We found that this is not the case: histones are in fact rapidly loaded onto MLV DNA immediately after delivery into the nucleus, well before integration [45,46]. We monitored the loading of histones onto viral DNA by Chromatin ImmunoPrecipitation (ChIP), in which infected cells are treated with formalin to cross-link proteins to DNA before lysis. The DNA is sheared by sonication, protein–DNA complexes are recovered by immunoprecipitation with specific antisera, and the DNA present in the precipitates is released and finally quantified by qPCR. The results showed that newly synthesized DNAs of retroviral vector genomes delivered by infection were rapidly associated with H3 and H2A core histones within 12 h after infection, and were recovered in immunoprecipitates at efficiencies comparable to that of host chromosomal DNA by 24 h post infection. The results were similar with PCR readouts of either total viral DNA, or with PCR readout of the LTR-LTR junction sequences present on 2-LTR circular DNA. The timing suggested that histone loading occurred before integration. Indeed, even when integration was blocked, either genetically by mutation of the viral integrase, or pharmacologically by the addition of an integrase inhibitor, the extrachromosomal viral DNAs still rapidly acquired histones [45].

Because MLV PICs cannot enter the nucleus of nondividing cells, it was possible to determine whether histone loading could take place in the cytoplasm or only after nuclear entry. Cells arrested in the G1/S phase of the life cycle by addition of aphidicolin were infected with retroviral vectors, and the viral DNAs were analyzed by qPCR. Cell fractionation showed that the viral DNAs were present at high levels in the cytoplasm and not in the nucleus, as expected, and that formation of the 2-LTR forms was almost eliminated. ChIP analysis showed a dramatic reduction in association of the viral DNA with histones, suggesting that nucleosome loading on the viral DNA occurs primarily—if not exclusively—after nuclear entry [45]. One more aspect of histone loading was revealed by study of MLV mutants. Successful MLV infection requires the functions of the p12 protein, a cleavage product of the Gag precursor, and specific p12 mutations block early events of the life cycle [47]. Infection by these p12 mutants resulted in normal reverse transcription to form PICs with linear viral DNA, but circular forms did not appear; the viral DNA did not accumulate in the nucleus or integrate into the host genome [48]. The key function of p12 was found to be the tethering of the PIC to host chromatin [49,50,51,52]. ChIP analysis showed that these mutant DNAs were not loaded with histones but instead retained association with capsid and nucleocapsid proteins [53]. These DNAs were not actively transcribed but remained silent. Thus, tethering of the PIC to host chromatin by p12 is required for normal uncoating, retention in the nucleus, histone loading onto the viral DNA, and expression. There is another setting in which nuclear entry by a retrovirus is blocked: infection of murine cells by the primate Mason-Pfizer Monkey virus is prevented by a failure of the PIC to enter the nucleus [54]. We examined M-PMV DNAs after infection of murine cells by ChIP, and here too the viral DNA showed highly reduced levels of association with histones. We concluded that when nuclear entry is blocked, histone loading does not occur [45].

We were then motivated to ask whether the histones loaded on unintegrated MLV DNAs in permissive cells were marked by the covalent modifications that are normally associated with silencing of heterochromatin. Indeed, they are. We used ChIP to show that the MLV DNA was associated with histones that were heavily marked by trimethylation of H3 tails on lysine 9 (H3K9me3, a marker of silenced chromatin). Knockdown of expression of SETDB1/ESET, the major histone methyl transferase responsible for H3K9 trimethylation, resulted in a substantial increase in reporter gene expression, and a modest reduction in the levels of the H3K9me3 mark on linear DNA, though not on the 2-LTR DNA. We did not see association of the viral DNA with histones bearing H3K27me3, another mark often associated with silent chromatin. We also could ask if the unintegrated DNAs were depleted of modifications such as H3 and H4 acetylation that promote transcription. We found that unintegrated MLV DNAs delivered by an integrase-defective virion were associated with histones with strongly reduced levels of acetylation, correlated with their poor expression. Treatment with TSA led to much higher levels of histone acetylation on the viral DNA, and much higher levels of expression. A surprising twist in the story was that TSA also led to higher accumulation of the unintegrated viral DNA over time—whereas the DNAs tended to disappear in untreated cells, they persisted at much higher levels when they were serving as templates for active transcription. Either the acetylated histones on the DNA, or the process of transcription, stabilized the unintegrated DNA and prolonged its half-life.

It is unclear whether histone deacetylation or histone methylation is the primary determinant of silencing. Since addition of TSA (to prevent deacetylation) can relieve the silencing, and since knockdown of SETDB1/ESET (to prevent methylation) can also do so, it may be that the two modifications act independently and additively. However, it may also be that deacetylation occurs first and promotes the H3K9 methylation. The strong effect of TSA on expression suggests that histone deacetylation is at least a major determinant of silencing. It further suggests that there is an ongoing dynamic acetylation and deacetylation cycle acting on the histones of the unintegrated DNA, and that in the presence of TSA, histone acetylation rapidly wins out. Many histone acetyltransferases (HATs) are known [55,56,57,58]. The identity of the histone acetyltransferases (HATs) responsible for viral gene activation is uncertain, but the most prominent HATs in most mammalian cells are the closely related CBP and p300 [59]; Tip60 and related proteins (members of the MYST family) [60]; and Gcn5 and PCAF (components of the SAGA complex) [61]. Various of these may be involved in activating expression in the presence of HDAC inhibitors and may also function to promote expression after viral DNA integration. The acetylation of histones may directly affect nucleosome mobility and allow access to transcriptional machinery. The acetylation mark is also recognized by the bromodomain of multiple “reader” proteins that remodel chromatin and activate transcription, including the BRD and SNF/SWI proteins.

These observations about the silencing of unintegrated viral DNA encouraged us to try to directly identify host factors that might mediate the process. We performed an unbiased genome-wide screen, using a CRISPR–Cas9 gene knockout (KO) library, to search for genes required for MLV silencing [62]. We generated a large pool of gene KO cells, and challenged these by infection with an integrase-defective MLV vector expressing the green fluorescent protein (GFP). The vast majority of the cells silenced the reporter, but rare cells lacking any gene essential for silencing would express GFP. We sorted the GFP-positive cells by FACS, and after culturing for a period to allow the viral DNA to decay, could repeat the challenge and again sort for GFP-positive cells. To identify the genes that had been targeted, we sequenced the guide RNAs encoded in the DNA of the cells selected for expression of unintegrated DNA. The most highly enriched gRNAs in the pool had targeted genes encoding five proteins: the histone methyl transferase SETDB1/ESET; all three subunits of the HUSH complex (MPP8, TASOR, and peripherin); and a new player, NP220. SETDB1 is the major HMT responsible for H3K9 trimethylation. The HUSH complex was originally identified as important in the silencing of proviruses integrated into heterochromatin [63], and in the silencing of retrotransposons [64]. NP220 is a nuclear double-stranded DNA (dsDNA)-binding protein with a preference for cytidine clusters, and containing a DNA-binding domain and a single C_2_H_2_-type zinc finger motif. We confirmed the role of these gene products by depleting them individually. Knockout or knockdown of *SETDB1*, but not other HMTs, relieved the silencing of unintegrated retroviral DNA. Knockout of any one of the three HUSH subunits similarly reduced the silencing of unintegrated MLV DNA, indicating that all three were required. Finally, knockout of NP220 also relieved the silencing. Re-expression of NP220, but not constructs lacking the DNA binding domain or the zinc finger, restored the silencing of the GFP reporter to the NP220 KO cells. We concluded that all these proteins were all needed to establish the silencing of incoming MLV DNAs.

**Figure 1:**
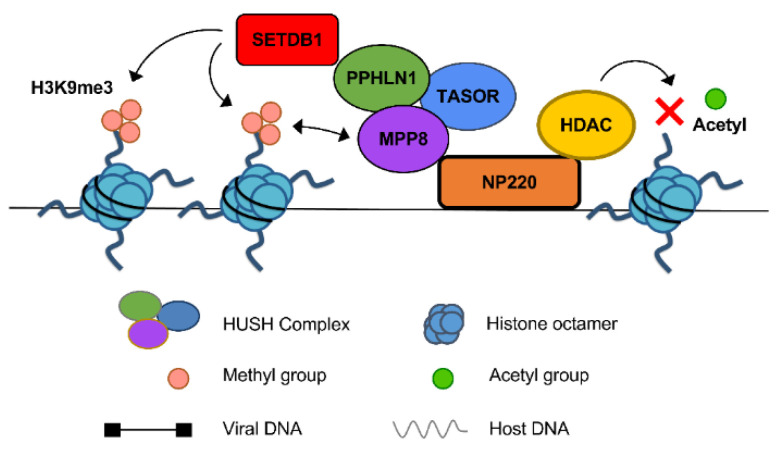
Silencing complex bound to unintegrated MLV DNA. The various proteins implicated in silencing incoming unintegrated DNA of the Moloney murine leukemia virus are depicted here. NP220 is bound directly to specific sequence elements on the viral DNA, and acts as a tether to bring histone deacetylases (HDACs) to remove acetyl groups from nearby histone tails, and to bring the trimeric HUSH complex through interactions with the MPP8 subunit. This in turn brings the SETDB1 histone methyltransferase to add the H3K9me3 mark to the H3 histone tails. MPP8 further binds the complex to the DNA by its interaction with the H3K9me3 mark. From [62].

Biochemical tests of the five genes provided some information about how they act to perform silencing. All the proteins were found to be bound to unintegrated MLV DNA by ChIP analysis. KO of any one of them caused the loss of all of them from the viral DNA, with one exception—NP220 alone was able to bind to the DNA in the absence of any of the others. This suggested that NP220 bound to the DNA first and served to bring the rest to mediate the silencing. The KOs of any of them also resulted in loss of the H3K9me3 marks on the viral chromatin, indicating that the complex was apparently needed to bring the SETDB1/SET methyltransferase to mark the viral histones. The presence of the mark likely served to tether the complex to the DNA in a positive feedback loop: the MPP8 subunit of HUSH is known to bind to H3K9me3, and indeed expression of a nonbinding mutant of MPP8 in an MPP8 KO line did not fully restore the presence of the complex on the DNA as assessed by ChIP. The KOs of any of the five also resulted in increased histone acetylation, correlated with the increased expression. We tested for the importance of any of a set of known HDACs that might be involved in silencing, and indeed knockdown of two of them—HDAC1 and 4—showed at least moderate relief of silencing. Both of these HDACs were bound to viral DNA as assessed by ChIP, both were lost in NP220 KO, and at least one of these (HDAC4) was found to strongly associate with NP220 by co-immunoprecipitation. Other HDACs might well also be involved. The picture that has emerged of the silencing machinery is shown in Figure 1.

Not all retroviral DNAs are equally silenced by this machinery, and the simplest explanation is that some viruses contain binding sites for the complex and some do not. The data suggested that NP220—known to prefer the sequence CCCCC(G/C)—might be the primary determinant of binding. We scanned the LTRs of MLV, HIV-1, and ALV for matches to this consensus and found five in MLV, six in HIV-1, and none in ALV. We generated a number of mutants of the MLV LTR with alterations in these binding sites, and found that the successive removal of these sites indeed rendered the virus increasingly nonresponsive to KO of NP220 [62]. The removal of the sites also resulted in reduction of the binding of NP220 to the DNA, as assayed by ChIP, as would be predicted. Similar results were seen with mutants of the HIV-1 LTR: removal of the consensus binding sites reduced the virus response to KO of NP220. As expected, based on its lack of binding sites, ALV was not affected by NP220 KO.

## 4. Silencing of Unintegrated HIV-1 DNA

While the silencing of incoming viral DNA expression is very common in many cells, we should not assume that all viruses will be treated similarly, or by the same machinery. Different retroviruses synthesize their DNA and deliver that DNA into the nucleus in very different ways, and are therefore exposed quite differently to the restriction systems acting in the cytoplasm and nucleus. For these reasons, it has been of interest to determine the properties of newly synthesized DNA formed by HIV-1 and compare them with those of the MLVs.

Unintegrated HIV-1 DNAs formed by integration-defective virions, or formed in the presence of integrase inhibitors, were found to be heavily silenced, much like those of the MLVs [65,66,67]. Examination of the presence of histones on the unintegrated HIV-1 DNA by ChIP revealed the rapid loading of histones H3 and H2B, as seen for the MLVs [65,66]. Moreover, analysis of the histones by ChIP again revealed the presence of marks associated with silencing: histones bound to the HIV-1 viral DNA were heavily marked by H3K9me3, and were very low in H3 acetylation. As with MLV, the HDAC inhibitor TSA led to strong activation of expression of unintegrated HIV-1 DNAs; and as with MLV, activating transcription with TSA led to an increase in the half-life of the unintegrated DNAs [65,66]. Tests showed the presence of at least two isoforms of the linker histone H1, namely, H1.2 and H1.4. These histones are typically involved in condensing nucleosomal DNA into higher-order structures and suggest that the HIV-1 DNA was likely present in a condensed state similar to heterochromatin. In addition, the H3 variant histone H3.3, argued to be involved in silencing of select endogenous retrovirus elements [68,69,70], was also found to be bound to the unintegrated HIV-1 DNA [65]. The presence of these variant histones on the viral DNA was not unique to HIV-1, but rather was found to be also true for MLV as well. Thus, the main features associated with silencing of MLV and HIV-1 DNAs are highly conserved.

Deeper analysis of the state of the histones on HIV-1 DNA, both unintegrated and after integration, has been exceptionally informative. Micrococcal nuclease digestion of chromatin after HIV-1 infection of Jurkat cells, followed by selection for viral DNAs and deep sequencing of the resulting DNA fragments, revealed a highly reproducible pattern of protected sequences [66]. The pattern was consistent with an ordered nucleosomal array along the viral DNA. Analysis of the DNA of wild-type virus at 9 h post infection showed nucleosomes positioned upstream of the transcriptional start site (one such dubbed Nuc0), one directly over the start site (NucDHS, for DNase hypersensitive site), and more further downstream (Nuc1, 2, 3, and more). At 48 h, when most of the DNA has been integrated, the pattern of protected fragments was very similar, but the nucleosome at the start site was largely lost, and Nuc0 and Nuc2 were slightly displaced in the upstream direction. Analysis of the DNA of an integrase-deficient mutant HIV-1 at 9 h after infection revealed a pattern nearly identical to the wild type, but that pattern remained unchanged at 48 h. Thus, the removal and repositioning of the nucleosomes required or occurred upon the integration of the DNA. The results suggest the rapid formation of an ordered, dense nucleosomal array on the silenced unintegrated DNA, and that integration results in rearrangements that correlate with activation of expression. Integration led to additional changes: ChIP analysis revealed the appearance of RNA polymerase II on the viral DNA, and an increase in H3 acetylation and H3K4 trimethylation. Notably, the sliding of the nucleosomes Nuc0 and Nuc2 was not observed in primary T cells, though all other features, including NucDHS eviction, seemed similar. The results suggest some differences between cell types in the machinery controlling nucleosomal arrangements.

Both MLV and HIV-1 are silenced by host factors that include SETDB1/ESET, and in spite of the many similarities between MLV and HIV-1 silencing, significant differences do exist. The most striking difference is in the response to KO of various components of the silencing machinery. While TSA inhibition of HDACs relieved the silencing of both viruses, KO of the HUSH subunits only relieved the silencing of MLV and had essentially no effect on silencing of HIV-1 [62,66,67]. If we think that one role for HUSH in the silencing of MLV is to bring SETDB1/ESET to the viral DNA to mediate histone methylation, and to tether the complex to H3K9me3 by the action of MPP8, then some other factor must perform these jobs in the case of HIV-1.

More silencing factors are being identified. One clue as their potential identities was the finding that the HIV-1 accessory gene product Vpr enhanced expression of unintegrated DNA [42,43]. Vpr typically acts by targeting many cellular substrates for polyubiquitinylation and proteasomal degradation, and indeed its relief of silencing required that Vpr interact with its E3 ligase partner Cul4A-DDB1 via the adapter DCAF1 [67]. A CRISPR–Cas9-based KO screen of a set of candidate Vpr target genes for any that might be required to silence unintegrated HIV-1 DNA identified SMC5-SMC6 complex localization factor 2 (SLF2) [67]. SLF2 is known to recruit the large SMC5/6 complex to sites of DNA damage [71], promoting repair at these sites. The KO of SLF2, or any of the six subunits of the SMC5/6 complex, resulted in a roughly two- to three-fold increase in expression of a reporter gene from unintegrated HIV-1 DNA. Depletion of other gene products involved in tethering the SMC5/6 complex to sites of DNA damage did not relieve the silencing. The complex may repress expression from many extrachromosomal DNAs. It is notable that the complex has also been shown to repress expression of the extrachromosomal DNA of Hepatitis B virus, and that the viral gene product HBx degrades SMC5 and 6 to relief that repression [72,73], in a close analogy to the degradation of SLF2 by HIV-1 Vpr. How does the SMC5/6 complex repress unintegrated DNAs? We do not yet know how SMC5/6 selects its targets for silencing, nor much about its mechanism of action. Interestingly, the KO of SLF2, or depletion by Vpr, did not result in changes in the levels of the H3K9me3 or H3K27me3 silencing marks on unintegrated HIV-1 DNA, but did lead to an increase in the H3K4me3 and H3 acetylation marks characteristic of active chromatin. This suggests that SMC5/6 is not simply inducing heterochromatin by adding silencing marks to histones. The SMC5/6 complex is known to bind DNA and mediate loop extrusion and chromatin compaction, and it may thus act directly on the viral DNA to alter its conformation. Consistent with this idea, assays for accessibility of the viral DNA to targeting by the Tn5 transposase (ATAC-seq) revealed an increase in accessibility upon SLF2 depletion. The exact function of the SMC5/6 complex in mediating the silencing remains to be determined, but it likely involves the compaction of chromatin structures.

The transcriptional regulators of some retroviruses can have profound effects on the silencing of unintegrated DNAs of distantly related retroviruses, such as HIV-1. A striking example is the activation of unintegrated HIV-1 DNA expression by the HTLV-1 Tax protein [74,75]. Tax is best characterized as transactivating expression of the HTLV-1 transcriptional promoter of the viral LTR, and its mechanism of action is the potent activation of the NF-kappa B transcription factor, through activation of the IKK1 and IKK2 kinases. This is achieved with the help of a ubiquitin E2 conjugating enzyme (UBC13) [76] and a ubiquitin E3 ligase, ring finger protein 8 (RNF8), promoting the auto-ubiquitinylation of Tax [77] and perhaps ubiquitinylation of other targets. Infection of cells expressing Tax by integrase-deficient HIV-1 genomes results in activation of NF-kappa B, and expression from the unintegrated DNA at levels sufficient to produce progeny virus and sustain a spreading infection in the culture [75]. Mutant HIV-1 genomes lacking the NF-kappa B binding sites were not responsive to this Tax-mediated activation. A very different method to promote expression from unintegrated DNA was achieved by insertion of an origin of DNA replication from the SV40 virus into an integrase-deficient HIV-1 genome and infecting Jurkat T cells expressing the SV40 large T antigen [78]. In this setting, the unintegrated DNA circles were maintained as replicating episomes at a high copy number without integration.

We note that not all cell lines or cell types will necessarily treat incoming DNAs in the same way. The magnitude of silencing of unintegrated DNA—that is, the ratio of expression of unintegrated over integrated DNA—is not identical in all cell types. While most cell lines in culture will repress unintegrated DNA over integrated DNA by about 5–10 fold or more, normalized to the amount of viral DNA per cell, some cells are remarkably more efficient. In particular, a number of lymphoid cell lines seem to be strikingly potent in silencing viral DNAs before integration but perfectly competent at expression after integration. Conversely, some lymphoid lines are quite inefficient at silencing an integrase-defective virus, and in these cells there may be integration mediated by host enzymes, thereby bypassing the need for viral integrase [44]. In another example, whereas we found strong effects of NP220 KD on expression of unintegrated HIV-1 DNA in the MT-4 T cell line, others have reported little or no effect of NP220 KO in 293T cells [75]. The bases for these various cell-type variabilities are not yet known.

## 5. Changes upon Integration

An intriguing aspect of the silencing of unintegrated DNA is that in permissive cells, retroviral DNA silencing is dramatically relieved by unknown mechanisms upon integration into the host chromosome. The integrated proviral DNAs, in most cases, is then highly activated to produce progeny virus. It is not obvious what is sensed upon integration, or precisely what changes, since the viral DNA sequences of course remain the same. It is very likely that the extent of activation depends on the state of the chromatin at the site of integration, but this connection is not completely clear. Integration into euchromatic regions (for MLV and HIV-1, the most frequent events) typically results in transcriptional activation, while integration into heterochromatic regions often results in silencing [63,64]. Insertions into euchromatin, and into active chromosomal regions, may represent the major sources of transcription of the provirus in a bulk infection. When viral DNA insertion is in a suitable location, the repressive proteins, and the repressive histone marks, both disappear after integration [45]. The activation, at least for HIV-1, is associated with eviction or rearrangement of the repressive nucleosome at the transcriptional start site, giving access to the transcriptional machinery and RNA polymerase II [66]. The relief from silencing does not seem to involve displacement of nucleosomes by host DNA replication fork movement through the provirus, because it is observed even with infection by lentiviral vectors in arrested cells [31]. The process may well involve spreading of active chromatin from flanking regions into the silent provirus, analogous to the spreading of inactive chromatin along DNA [79,80,81,82]. However, it may involve more complex changes that are triggered upon integration. Integration does cause transient DNA damage—single-strand nicks and small gaps that are rapidly repaired [83]. The damage may be sensed and trigger the association of proteins such as the histone H2AZ, and these events may promote nucleosomal rearrangements or changes in chromatin state.

Those retroviruses that encode trans-acting transcriptional activators, such as the HIV-1 Tat protein, enjoy a strong positive feedback loop, such that initial low-level expression drives higher expression, and this may play a role in overriding any residual silencing of the pre-integrative DNA. However, other viral proteins may also promote the transition to active expression. We recently found that the HIV-1 integrase (IN) has such activity [84]. The HIV-1 IN protein is subject to multiple covalent post-translational modifications, including acetylation of multiple lysine residues in the C-terminus. Mutations altering these lysines do not prevent integration but have the interesting effect of delaying the activation of expression after integration. The result indicates that the wild-type IN normally plays a role in jump-starting transcription immediately after integration, promoting the switch from silent to active provirus. The mechanism remains unclear but would most likely involve the recruitment of factors through the C-terminal tail of integrase.

## 6. Silencing after Integration

While infection of permissive cells typically results in high-level expression of the integrated proviral DNA and rapid production of progeny virus, infection of particular cell types results in a very different outcome. In embryonic stem (ES) cells, hematopoietic stem cells, and other developmentally primitive cell types, the proviral DNAs at nearly all insertion sites are more uniformly silenced. The cellular machinery that mediates this silencing seems to differ from that used in silencing unintegrated DNA, though there is some overlap. The silencing of MLV in mouse ES cells is initiated by sequence-specific DNA-binding proteins (ZFP809 and YY1) that recognize targets on the viral DNA and bring a large protein complex to effect silencing [85,86]. The central component of the complex is TRIM28/Kap1, which acts as a scaffold to recruit histone deacetylases, the histone methyltransferase SETDB1, the methylated histone binding protein HP1, and ultimately DNA methyl transferases [87,88]. Repressive marks remain on the histones, and the DNA becomes methylated on CpG residues [89]. Screening genome-wide siRNA libraries has revealed a large number of additional genes that are required for this silencing [90]. The silencing of avian sarcoma virus (ASV) genomes in mammalian cells involves another array of host proteins—including distinctive ones—that perform similar tasks [91], with the histone chaperone DAXX playing a central role, in part by interacting directly with the avian integrase (IN) [92,93]. Much of this silencing machinery plays a critical role in repressing the vast array of endogenous retroviruses in the cell genome.

The establishment and maintenance of silent HIV-1 proviruses in HIV-1-infected patients is the major barrier to the development of a true cure for AIDS. Chronic infection results in the accumulation of long-lived memory T cells with silent proviruses, constituting a latent reservoir of viral DNAs that can be activated at any time to initiate the production of progeny virus. This silencing and activation is controlled by histone modifications [94] and DNA methylation, likely established in part by the chromatin status at the sites of provirus integration [95]. The hope is that manipulating the writers and readers of these modifications could allow either for the complete activation of the silent proviruses (the “shock and kill” strategy) or their permanent silencing (the “block and lock” strategy). Neither approach is certain of success.

## 7. Conclusions

The silencing of incoming DNAs is a defense system found in a wide range of cells of many species, reflecting the ongoing battle between virus and host. The system might be most important for the DNA viruses, where strong silencing might be capable of actually preventing infection. The potential significance of the block is perhaps reflected in the evolutionary pressure it imposes on DNA viruses, made apparent by the presence of viral genes such as the herpes virus ICP0 gene, which inactivate the silencing machinery and allow early gene expression. How important is the silencing of unintegrated DNA to MLV, HIV-1, or to any retrovirus? It might be argued that there is only a small window of time when unintegrated DNAs are present, and that viral expression from the provirus formed after integration is overwhelmingly the major source of viral mRNAs and proteins. Indeed, virus replication in cell lines engineered with NP220 KO, relieving the silencing, was only slightly accelerated, with growth curves of spreading infections shifted only a day or two earlier than in control cell lines [62]. Nevertheless, keeping the virus silent as much as possible may help delay the spread of virus, and could also reduce the efficiency of virus transmission from host to host.

Retroviruses certainly function well in spite of the silencing activity of the host. Indeed, there is an argument that keeping the genome “quiet” until after integration may be advantageous for retroviruses: silencing may allow the virus to escape detection until it is too late for the cell to block the establishment of a permanent source of progeny virus. In this regard, retroviruses benefit from the fact that they can enter a cell, carry out reverse transcription of their RNA to form unintegrated DNA, and integrate the DNA to form the provirus, all mediated by proteins imported with the virion, and without any need for viral gene expression in the infected cell. Vigorous expression can follow after integration when there is no chance for the cell to eliminate the viral DNA. Silencing of incoming DNA may benefit the virus in another way: it may enhance the ability of the virus to establish a chronically silent provirus in the pool of latently infected cells. The rare cells that acquires a silent provirus will never have expressed any viral gene products and so will raise no warning flags to the host immune system that they now carry a dangerous program—a literal time bomb—that can be activated at any time in the future to initiate a fatal viremia. We will need to understand much more about the regulation of viral expression to be able to deal with retroviral latency.
